# Editorial zur Special Section „Neuer Antisemitismus?“

**DOI:** 10.1007/s41682-022-00114-3

**Published:** 2022-05-09

**Authors:** Oliver Hidalgo, Philipp W. Hildmann

**Affiliations:** 1 Institut für Politikwissenschaft, Universität Regensburg, Regensburg, Deutschland; 2 Kompetenzzentrum Gesellschaftlicher Zusammenhalt und Interkultureller Dialog, Hanns-Seidel-Stiftung, München, Deutschland

Der Antisemit Carl Schmitt (Schmitt 2010, S. 87) schrieb einst im thematischen Umkreis seiner ‚Politischen Theologie‘, dass der Feind „unsere eigne Frage in Gestalt“ sei. Demnach stellt der Feind nicht allein die Existenz einer bestimmten kollektiven Identität in Frage. Entlang von Feindbildern und Sündenböcken lassen sich überhaupt erst die Denk- und Argumentationsmuster generieren, um ein ‚Wir‘ in Abgrenzung von einem ‚Anderen‘ bzw. einem ‚Fremden‘ zu identifizieren.

Die negativen Stereotypen, Pauschalverurteilungen und Diskriminierungen, denen Juden seit mehr als 2000 Jahren ausgesetzt sind und die, wie das Attentat von Halle im Oktober 2019 in schockierender Weise verdeutlichte, nach wie vor in blinden Hass, Mord und Verfolgung umschlagen können, haben sich dieser Lesart zufolge fest in das gruppenspezifische Selbstverständnis der europäischen Gesellschaften eingebrannt. Die Formen des Antisemitismus mögen dabei zwar einem permanenten Wandel unterliegen, das Phänomen als solches aber ist in all seiner Veränderlichkeit als konstant anzunehmen. Vom religiös geprägten Antijudaismus über den modernen antisemitischen Rassismus und dem danach wirkenden Verdrängungsmechanismus im sekundären Antisemitismus bis hin zum stark israelbezogenen Antisemitismus der Gegenwart – immer waren Jüdinnen und Juden Ziel von Abwertung, Ausgrenzung, Ressentiments und Feindseligkeit. Umso wichtiger ist es aus Sicht der *Zeitschrift für Religion, Gesellschaft und Politik* (ZRGP), die offenen wie latenten Erscheinungsbilder, die der Antisemitismus als politische Ideologie, Legitimationsressource von Gewalt sowie als identitätsstiftende Markierung von Freund-Feind-Dichotomien angenommen hat, kontinuierlich einer kritischen, sozialwissenschaftlich und zeithistorisch imprägnierten Reflexion zuzuführen.

Die in der vorliegenden Special Section versammelten Beiträge, die bis auf eine Ausnahme allesamt auf Vorträgen im Rahmen einer Fachtagung beruhen, die der AK Politik und Religion der DVPW in Kooperation mit der Hanns-Seidel-Stiftung am 17./18. September 2020 in Kloster Banz veranstaltet hat, stehen zum einen im Zeichen der schwierigen Debatte um den ‚neuen‘ Antisemitismus, die sich in der Öffentlichkeit wie der Wissenschaft in den letzten Jahren zugespitzt hat (siehe Heilbronn et al. 2019). Dabei ist gar nicht einmal strittig, dass sich im Antisemitismus der Gegenwart tradierte Formen mit ‚neuen‘ Aspekten und Komponenten vermischen, so etwa das (rechts- wie linksextreme) Feindbild der Juden als treibende Kraft eines zerstörerischen Kapitalismus, der von der Globalisierung weiter angestachelte Mythos der jüdischen Weltverschwörung oder auch der Antijudaismus in bestimmten Episoden in Koran und Sunna, der durch die politische Ideologie des Islamismus im 20./21. Jahrhundert lediglich an Bedeutung gewonnen hat (Botsch 2019). Kontrovers diskutiert wird stattdessen, inwieweit sich der ‚neue‘, vom Israel-Palästina-Konflikt dominierte Antisemitismus tatsächlich durch eine zumindest der Tendenz nach alternative Trägerschaft auszeichnet, bei der arabisch-nationalistische, islamistische und linksradikal-antizionistische Kräfte in den Vordergrund rücken, während die traditionell antisemitischen Milieus im Rechtsextremismus, Faschismus und christlichen Fundamentalismus bzw. Konservatismus an Bedeutung verlieren (Laqueur 2008). Damit verbunden ist die heftig polarisierende Frage, ob die im ‚neuen‘, sekundären ‚Entlastungs-Antisemitismus‘ angelegte Täter-Opfer-Umkehr, die vor allem zur Relativierung begangener Verbrechen sowie zur Leugnung einer historischen Schuld und Verantwortung gegenüber Jüdinnen und Juden lanciert wird, eine besonders besorgniserregende Dimension der Judenfeindlichkeit umfasst, die sich zunehmend in der ‚Mitte‘ der Gesellschaft konstelliert, oder ob dieses Argument seinerseits Gefahr läuft, zur Immunisierung der israelischen Siedlungspolitik gegenüber dem Grund nach berechtigter Kritik instrumentalisiert zu werden. Zu dieser Debatte will die nachfolgende Special Section relevante Bezüge herstellen sowie eigene signifikante Beiträge leisten.

Zum anderen wurden die hier publizierten Artikel unter dem fatalen Eindruck konzipiert und verfasst, dass im Kontext der Corona-Pandemie ein nahezu ‚klassisches‘ Verschwörungs- und Sündenbockdenken, das Jüdinnen und Juden bzw. dem Staat Israel in unterschiedlicher Manier die Hauptschuld an der Verbreitung, (angeblichen) Erfindung oder auch kommerziellen Ausschlachtung des COVID-19-Virus zuweist, eine zuvor kaum für möglich gehaltene Renaissance erlebt. Die befremdliche Präsenz, die etwa die offen antisemitische, aus den USA stammende neo-apokalyptische QAnon-Bewegung (Nagel 2021, S. 64–73) seit 2020 in Deutschland entwickelte, als zunächst zahlreiche deutschsprachige QAnon-Gruppen im Internet entstanden, die sich im Anschluss bei mehreren Protesten und Demonstrationen gegen die Corona-Maßnahmen mit Rechtsextremen, Reichsbürgern und rechten Esoterik:innen vermischten, ist in diesem Zusammenhang keinesfalls zu bagatellisieren (z. B. Beuth et al. 2020; Blume 2020; Pöhlmann 2020).

Zu Beginn der Section dokumentieren die Beiträge von *Samuel Salzborn* und *Robert Sigel* die langfristigen historischen Entwicklungslinien und Trends des Antisemitismus in Deutschland und Europa (ausführlich: Salzborn 2019) respektive die Kontroverse um die Arbeitsdefinition von Antisemitismus, welche die *International Holocaust Remembrance Alliance* (IHRA) 2016 beschlossen hatte und die in jüngerer Zeit zu verschiedenen Gegenentwürfen (u. a. der *Jerusalem Declaration* von 2021) führte. Beide Stücke besitzen den Charakter eines einführenden Überblicks und haben daher nicht das übliche Peer Review-Verfahren der ZRGP durchlaufen.

*Axel Töllner* zeigt anschließend in seinem Beitrag anhand einiger ausgewählter Beispiele auf, wie sich die Tradition des christlichen Antijudaismus auch noch im modernen, rassistischen Antisemitismus niederschlägt und wie trotz verschiedener Bruchstellen und Transformationen die antisemitischen Chiffren aus der (religiösen) Vergangenheit z. T. bis in die säkulare Gegenwart fortwirken. *Oliver Hidalgo* rekonstruiert daraufhin noch einmal die bereits skizzierte verfahrene Debatte um den ‚neuen‘ israelbezogenen Antisemitismus, um vor diesem Hintergrund den Versuch zu unternehmen, jene Debatte mithilfe von demokratietheoretischen Argumenten analytisch zu versachlichen. Die Sackgasse, in die sich der Diskurs seit einiger Zeit manövriert hat, sollte dadurch vermieden werden.

An diese beiden theoretisch-ideengeschichtlich argumentierenden Beiträge knüpfen zwei empirisch gehaltene Kurzstudien an. Dabei gehen *Cemal Öztürk* und *Gert Pickel* der Frage nach einem möglichen „Import“ von antisemitischen Einstellungen durch Einwanderer:innen aus Ländern mit muslimischer Mehrheitsbevölkerung nach. Ihr differenziertes Ergebnis bestätigt zwar einerseits die Verbreitung eines religiös aufgeladenen, israelbezogenen Antisemitismus unter Muslim:innen in Deutschland, betont jedoch andererseits, dass eben diese Variante des Antisemitismus im Vergleich zu den meisten Ländern der islamisch-arabischen Welt abgeschwächt ist (was auf Integrationserfolge hindeuten könnte), um zugleich zu ergänzen, dass der sekundäre schuldverleugnende Antisemitismus unverändert die Domäne rechter politischer Milieus bzw. der Bevölkerung ohne Migrationshintergrund bedeutet. Ein Autorenkollektiv um *Gert Pickel, Selana Tzschiesche, Katrin Reimer-Gordinska* und *Oliver Decker *widmet sich hingegen speziell dem Standort Berlin sowie der dortigen Existenz antisemitischer Ressentiments. Mithilfe eines triangulativen Ansatzes gelingt den Autor:innen diesbezüglich der Nachweis, dass die pluralistische Gesellschaft in Berlin mit einem vergleichsweise hohen Anteil jüdischer Bürger:innen weniger anfällig für Antisemitismus ist als andere Großstädte, wenngleich das Problem auch hier keineswegs gebannt sei.

Zum Abschluss der Special Section werden ergänzend zwei hochaktuelle europäische Perspektiven vorgestellt. Dies betrifft zunächst *Teresa Nentwigs* Untersuchung der Entwicklung des Antisemitismus in den Reihen des französischen *Rassemblement National* unter Marine Le Pen, die sich vom früheren christlichen Fundamentalismus des *Front National* unter der Ägide ihres Vaters Jean-Marie Le Pen lediglich offiziell losgesagt habe. Der Aufsatz von *Magdalena Marsovszky* beleuchtet abschließend die völkische Esoterik in Ungarn als eine die dortige politische Kultur beherrschende ‚Alltagsreligion‘, die unter dem Orbán-Regime zur zentralen Ressource einer Transzendentalisierung der Nation avancierte und zugleich als prägnante, zur staatsrechtlichen Grundlage erhobenen Manifestation einer antisemitischen Schuldabwehr gelten kann. Die von der Regierung Orbán selbst beschworene christlich-demokratische Identität Ungarns befindet sich dazu in einem krassen Gegensatz.

Zwei weitere Beiträge zum Antisemitismus im zeitgenössischen Rechtsextremismus sowie zu antisemitischen Einstellungen bei Christ:innen und Muslim:innen, die Gegenstand der Fachtagung im September 2020 waren, konnten bis zur Deadline der Special Section leider nicht fertiggestellt werden. Die Gastherausgeber der Special Section sowie die Herausgeber der ZRGP hoffen, dass diese beiden Studien eventuell zu einem späteren Zeitpunkt als Collection den eben vorgestellten Artikeln hinzuzufügen sind. Dies wäre für die ZRGP nicht zuletzt deswegen wichtig, da die teilweise verbreitete Auffassung, der ‚neue Antisemitismus‘ komme weitgehend ohne (traditionelle) religiöse Bezüge aus, zu vordergründig sein könnte. So konstatierte der Unabhängige Expertenkreis Antisemitismus des Deutschen Bundestages in seinem Bericht (2017, S. 184) eine folgenschwere „Diskrepanz zwischen den offiziellen Verlautbarungen beider Kirchen und den Einstellungen an der Kirchenbasis bzw. auf Gemeindeebene“, die es beim Kampf gegen den Antisemitismus im Auge zu behalten gilt. Gleichzeitig ist nicht zu unterschätzen, dass sogar auf diesem Sektor weniger genuin religiöse Überzeugungen am Werk sein könnten, als vielmehr vorhandene autoritäre Einstellungsmuster religiös verbrämt werden. Unabhängig davon sollten neue Studien die Tradition des christlichen Antijudaismus zur Erklärung existenter antisemitischer Züge bei schismatischen Glaubensgemeinschaften und ultrakonservativen innerkirchlichen Oppositionsgruppen (z. B. die Piusbrüder oder auch bestimmte Gruppen der Evangelikalen) stärker ins Visier nehmen als in der Vergangenheit. Auch eine intensivierte Betrachtung des Antisemitismus, der womöglich in den christlich-orthodoxen Kirchen oder auch in einigen freikirchlichen Gemeinden oszilliert, könnte einen lohnenswerten Forschungsgegenstand abgeben (ebd.).
